# Grafting Starch with Acrylic Acid and Fenton’s Initiator: The Selectivity Challenge

**DOI:** 10.3390/polym16020255

**Published:** 2024-01-16

**Authors:** Inge-Willem Noordergraaf, Judy R. Witono, Hero J. Heeres

**Affiliations:** 1 Faculty of Science and Engineering, Green Chemical Engineering, Groningen University, Nijenborgh 4, 9747 AG Groningen, The Netherlands; h.j.heeres@rug.nl; 2 Chemical Engineering Department, Parahyangan Catholic University Bandung, Jl. Ciumbuleuit no. 94, Bandung 40164, Indonesia; judy@unpar.ac.id

**Keywords:** starch graft copolymerization, acrylic acid monomer, graft selectivity, Fenton’s initiator, graphical representation of different grafting systems, water solubility of the monomer as a key factor, methods to improve selectivity, dedicated monomer dosage

## Abstract

Through the graft polymerization of acrylic monomers onto starch, materials with interesting new properties can be synthesized. Fenton’s chemistry, Fe^2+^/H_2_O_2_, is considered to be attractive for the initiation of graft polymerization with the monomer acrylic acid since it is cheap and reacts quickly at ambient conditions and should therefore be easy to scale up. However, the selectivity of the grafting versus the homopolymerization reaction poses a challenge with this monomer and this type of initiator. In the present review paper, we investigate why data from the literature on grafting systems with other monomers and initiation systems tend to show higher graft selectivity. A scheme is presented, based on reaction engineering principles, that supports an explanation for these observed differences. It is found that more selective activation of starch is a factor, but perhaps even more important is a low monomer-to-starch ratio at the starting sites of graft reactions. Since water is the most common solvent, monomers that are less water-soluble have an advantage in this respect. Based on the proposed scheme, methods to improve the graft selectivity with Fenton’s initiator and acrylic acid are evaluated. Most promising appears to be a method of gradual monomer dosage. With gelatinized cassava starch in a batch reactor, both the grafting percentage (17 => 29%) and graft selectivity (18 => 31%) could be improved. This can be considered a principal breakthrough. Still, more research and development would be needed to refine the method and to implement the idea in a continuous reactor at a larger scale.

## 1. Introduction

Graft copolymerization onto starch is an attractive option to synthesize new products from this renewable raw material. The principle of graft polymerization involves the addition of newly synthesized chains of functional polymer to the existing backbone, thereby imparting the improvement of existing properties or introducing completely new functionalities. In the past half-century, the topic of graft polymerization has been covered by many research groups around the world, as demonstrated by the large number of publications. There are relatively recent reviews by Meimoun et al. [1] and Lele et al. [2], as well as older reviews, e.g., from Athawale and Rathi [3], and from the superabsorbent pioneers of the US Department of Agriculture, Fanta and Doane [4]. Together, these reviews give a good overview of the many possible monomers, graft copolymerization initiators, and starch types, as well as of the influence of many reaction variables. The effects of reaction variables and the various types of initiation methods on graft selectivity are discussed in more detail in Section 4.1. The present review provides a somewhat different approach compared to the other reviews. We do not intend to make a full inventory of grafting systems and initiation methods. Instead, we address the factors that are important for the realization of good selectivity in starch grafting, based on data from the literature and results from our own research. 

Starch grafting has been the subject of research at Groningen University for over two decades now. Results from the first decade were reported in the PhD thesis of Judy Witono [5]. More publications will be cited further on, related to relevant discussion topics. Early on in the project, we selected acrylic acid as a monomer since, when graft-polymerized onto starch, water-soluble polymers can be synthesized with a large spectrum of possible applications. Such polymers are characterized as ‘performance polymers’. Potential applications of water-soluble performance polymers include viscosifiers or thickening agents, flocculation agents, and heavy-metal-adsorbing molecules, the latter two both for wastewater treatment [6]. Starch grafted with polyacrylic acid (designated as SgPAA) can also be applied as the cobuilder in detergent formulations and as a textile sizing agent. A separate class of applications is superabsorbent materials that can be used in diapers, hygiene products, wound dressings, for agricultural purposes such as water management in arid soils, and more. Unlike in the other applications mentioned, superabsorbent materials need to be water-insoluble. Such materials consist of a network of interconnected hydrophilic chains that can absorb huge amounts of water [7]. All of these various applications come with specific demands on the properties, which, in turn, put demands on molecular structural parameters like the size and spacing of the grafts or, for superabsorbents, the degree of crosslinking. In a previous review, we presented an overview of the application-related demands and how reaction variables can be used to meet them [6]. 

To synthesize water-soluble polymers from starch, we investigated a direct grafting route starting with the water-soluble monomer acrylic acid, with water as the logical reaction medium. Also, we selected Fenton’s reagent (Fe^2+^/H_2_O_2_) as the method of initiation, because it is cheap and reacts quickly under ambient conditions. Fenton’s reagent can be designated as an indirect method of initiation since it involves two steps: first, the creation of radicals, and then the transfer of the radicals to starch. Other initiation methods, such as direct starch activation, are discussed in Section 4.1. Cassava starch was used in most experiments and always thermally gelatinized before the start of the grafting reaction [5,8]. Together, these reagents give a homogeneous reaction system that should be relatively easy to scale up.

**Figure 1:**
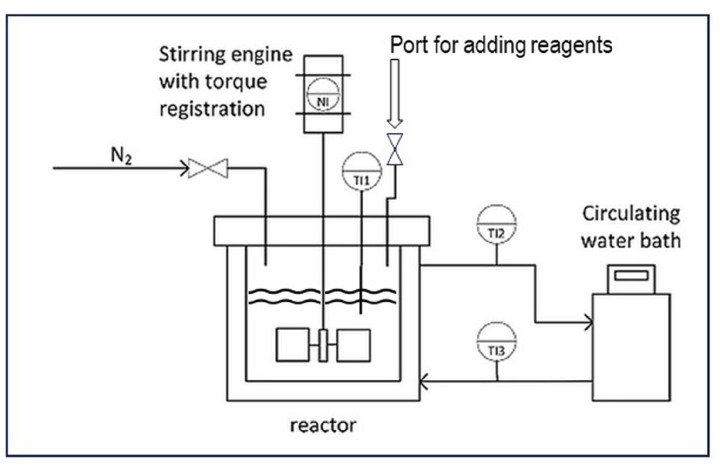
Drawing of the batch reactor for graft polymerizations.

The present review article is focused on the topic of graft selectivity, which an important factor in every grafting reaction with acrylic monomers and starch. Good graft selectivity means that the formation of the less-wanted byproduct homopolymer (polyacrylic acid in our case) is minimized. The factor graft selectivity, or GE (Figure 1), is dependent on many variables. In this paper, the influence of important variables on graft selectivity is discussed in a comprehensive way, based on data from the literature and on results from our previously reported research. We present a graphical schematic overview, in terms of reaction engineering considerations, of different grafting systems with respect to the water solubility of the monomer and the (in principle) different initiation methods. This graphical representation is useful to explain why a grafting reaction with indirect initiation and a water-soluble monomer poses the greatest challenge in the realization of good graft selectivity. Based on the theoretical insights thus obtained, several methods will be discussed to improve the selectivity of the grafting of acrylic acid from starch with Fenton’s initiator. Results from dedicated experiments are presented to assess the potential of such improvement ideas. 

In theory, homopolymer formation could also be avoided by methods involving controlled radical polymerization (CRP). In the review of Meimoun et al. [1] and in our review from the same year (2018) [6], atom transfer radical polymerization (ATRP) and reversible addition–fragmentation chain transfer (RAFT) polymerizations were assessed with respect to use of these methods in graft polymerization with starch. The conclusion was that these methods may hold long-term promise, but even then, CRP methods would be more suitable for products with a high added value and for production at small scale. A more recent update by Parkatzidis et al. [9] gives no reason to change that supposition. Therefore, in the present paper where starch grafting is considered with applications at a larger scale, we focus on conventional free radical polymerizations. 

## 2. Experimental Procedures 

**Figure 2:**
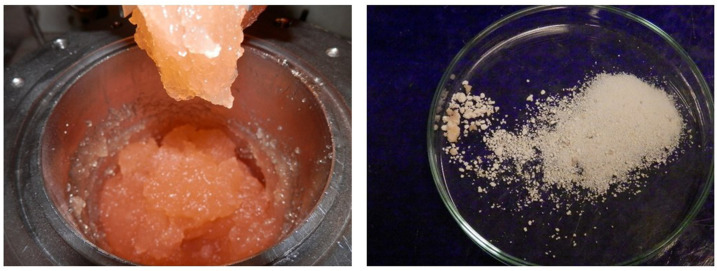
A picture for a visual impression of the gelly mass obtained from a standard reaction run (**left**) and of the grafted starch after separation and drying, before grinding for the instrumental analysis (**right**).

**Figure 3:**
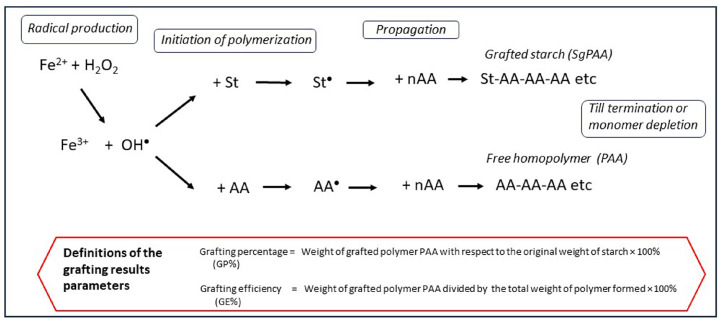
Reaction scheme of grafting versus homopolymerization with Fenton’s reagent and the important result parameters. St = starch, AA = monomer acrylic acid, • = radical state, PAA = polyacrylic acid.

Most of the experimental results reported in previous publications [5,8,10], as well as the experimental explorations of several selectivity improvement ideas reported in Section 6, have been obtained from grafting reaction runs in the stainless-steel bench-scale batch reactor depicted in Figure 1. The reactor is fitted with a water jacket connected to a circulating water bath, which has the function of a thermostration system. There is a dosage port for the reagents and a nitrogen purge to exclude molecular oxygen from the reaction environment. The overhead stirrer with an integrated torque meter allows for the monitoring of viscosity changes throughout the processes. Cassava starch is typically produced in large quantities in tropical areas and has been used in most of these experiments [5,8]. An exploratory study on the influence of reaction variables has been published, with the full details of the experimental system and the reagents [5,8]. From that assessment, a set of typical conditions for a basic (‘standard’) reaction procedure was chosen. Such a standard reaction run is preceded by the thermal gelatinization of cassava starch, at a dosage of 5–10 wt% in water, at a temperature of 70 °C, for 25 min. The mixture is stirred at a constant speed of 300 rpm throughout the process. After gelatinization, the reactor is cooled to the selected reaction temperature of 40 °C. The monomer acrylic acid is added at various concentrations via the port shown in Figure 1 and allowed to mix for several minutes. First, ferrous ammonium sulfate (FAS), the Fe^2+^ part of Fenton’s, is added and also mixed for five minutes before adding the hydrogen peroxide. A molar ratio of Fe^2+^ to AGU of 1:100 and a molar ratio of H_2_O_2_ to Fe^2+^ of 10:1 are used in most experiments [5,8,10]. The polymerization reactions start immediately after the addition of hydrogen peroxide, as discussed in Section 3.1. The reaction product is subjected to the analytical procedure as described in detail before [11]. The separation of homopolymer PAA from the sticky gel as shown in the picture of Figure 2 (left) is a key element in the procedure. A thorough separation is achieved by repeated washing with acetone. The separated homopolymer is quantitatively analyzed with HPLC, while ^1^H-NMR is applied to measure the amount of grafted polymer on the starch [11]. In the box inside Figure 3 the most important result parameters are defined. Grafting percentage or GP% is the amount of newly synthesized polymer attached to the starch backbone. GE% represents the selectivity of grafting versus homopolymer formation. Monomer conversion is another important result, which is also measured with HPLC [11]. Since in previous work, the monomer conversion proved to be 98–100% in almost every reaction [5,8], in the evaluation of more recent experiments the HPLC analysis has been skipped and GE% is calculated with the assumption of full conversion of the monomer. The formulas and the calculations of the results that are presented in Section 6.4 are given in the Supplementary Material. 

## 3. Grafting Reactions: The Selectivity Challenge

### 3.1. Grafting and Homopolymerization Reactions

In the scheme of Figure 3 it can be seen that radicals are created from the reaction of Fe^2+^ with H_2_O_2_. The highly active OH radicals can react with anhydroglucose units (AGUs) of starch by abstracting a hydrogen atom from a hydroxyl group and thus create a reactive radical site on the starch backbone. When a monomer molecule like acrylic acid (AA) encounters the starch radical, the radical reacts with the C=C bond and the monomer molecule becomes covalently attached to the backbone. The active radical shifts to the acrylic acid molecule that will in turn react with the next monomer, thus initiating the growth of a grafted polymer chain. The exact chemistry involved has already been depicted [5,8]. The reaction proceeds according to the principle of add-on polymerization [12], referred to as the propagation stage. Polymerization reactions usually end by termination between two radicals [12]. In some cases, polymerization ends by disproportionation, which is not only less common [12] but also not likely in this case considering the results shown in Section 3.2. By using Fenton’s there are two steps involved in the initiation of the grafting reaction: first, OH^•^ radicals are created in the solution, and the second step is that these radicals react with starch. We call this indirect initiation to distinguish this type of two-step initiation from methods that produce radicals via a single step and involve a direct reaction with starch (see Section 4.1). 

Figure 3 also shows the most important side reaction. Free hydroxyl radicals are created more or less at random in the solution and can react directly with monomer molecules and start another propagation chain, forming the homopolymer polyacrylic acid. This ‘choice’ is the perhaps most decisive moment that determines the initial selectivity, initiation of grafting versus initiation of homopolymerization. The very principle of the radicals being created in the solution of a homogeneous reaction system means that this side reaction cannot be avoided. So, in this system there will always be homopolymer formation. In principle, this is a waste of monomer and, also, separation of the physically entangled chains of homopolymer and grafted polymer may be difficult—see the picture of Figure 2. For the purpose of product characterization, we previously reported an elaborate method for efficient separation of the homopolymer and grafted polymer based on repeated washing with acetone [11] (Section 2). To apply such a separation in larger-scale production is not very economical as it will use large amounts of solvent. However, in some potential applications the presence of some homopolymer in the product may be acceptable, for example, in viscosifiers, flocculation agents, or in textile sizing agents [6]. Even if its presence is tolerable, homopolymer does not contribute much to or may even negatively affect the properties of the product, like in superabsorbent applications [7]. Thus, it is clear that a high as possible graft selectivity should be striven for.

### 3.2. Initial and Final Graft Selectivity

**Figure 4:**
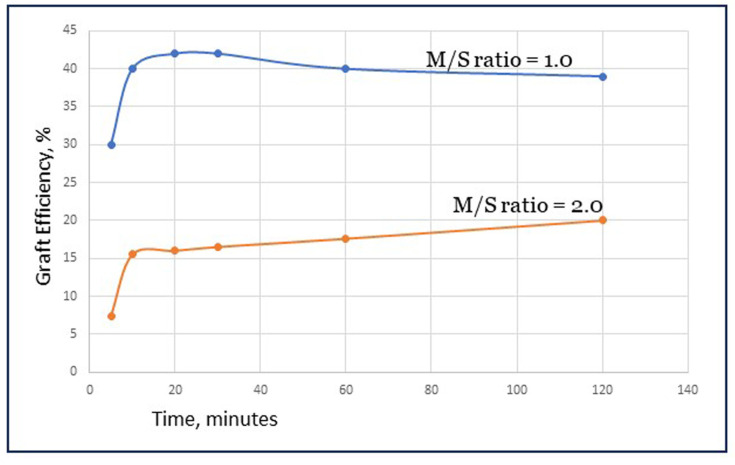
The course of the graft selectivity during the reaction, at two moleculare ratios of monomer to starch (M/S).

The ratio of OH^•^ radicals that reacts either with starch or with monomer determines the selectivity of the reaction in the early stage. This ‘initial selectivity’ is a matter of statistical probability. The initial selectivity can be influenced by reaction conditions, for example, by the relative concentrations of the AGUs in the starch backbone versus AA present in the vicinity of the activated starch site. However, the initial selectivity may not be the only factor that determines how much grafted starch versus homopolymer is formed at the end of the process. It can be imagined that growing chains of grafted polymer can terminate not only with another growing graft but, alternatively, with a radical end group of a growing homopolymer chain. We can designate the latter as crosstermination. Whether this happens is also a matter of probability, in which a factor like the mobility of the chains in a viscous solution may play a role. GE data that are reported in the literature are usually the values obtained at the end of the reaction. This makes it impossible to know if there is a contribution of crossterminations to the final selectivity. Calculations based on data reported by Witono et al. [5] give a rather unique view on how the parameter GE evolves during some typical reactions, as is shown in Figure 4. The calculations are in the Supplementary Material. These data points may have some margin of error since they are derived from combining several experimental data but certainly allow for a trend analysis. In Figure 4, it can be seen that GE rapidly increases in the first minutes of the reaction, then levels off or even slightly decreases. But, in the end, the GE% is always higher than in the first minutes. With Fenton’s initiator, there is also a slow second step of radical creation from ferric ions (Fe^3+^) [10,13]. It is uncertain if HO_2_^•^, the radicals created in that step, are sufficiently reactive to also start grafting reactions, but that is possible since it has been demonstrated that they do react with starch [10]. This can be a contribution to higher graft selectivity according to the following. If these radicals indeed do start new grafted chains, that occurs later on in the reaction when part of the initially fed monomer has been consumed. Then, there is a lower actual ratio of monomer to starch (M/S), which is beneficial for graft selectivity, see also Section 4.2. However, when this effect can be considered minor, the course of the GE lines in Figure 4 is mainly caused by crossterminations, and those may indeed contribute to a higher ‘final selectivity’. The increment of GE during the reaction is higher when there is more monomer in the system (at M/S = 2). This is consistent with the effect of statistical probability since, then, there will also be more growing homopolymer chains that a grafted chain can meet to terminate with. The examples in Figure 4 also show that, at the lower M/S ratio of 1.0, GE% is higher both at the end and in the early stage of the reaction. To conclude, crossterminations may compensate to some extent for a low initial selectivity, but the overall grafting result will be better when the selectivity at the initiation stage (initial selectivity) is higher. 

## 4. Factors That Influence the Graft Selectivity 

### 4.1. The Choice of the Initiator System

**Figure 5:**
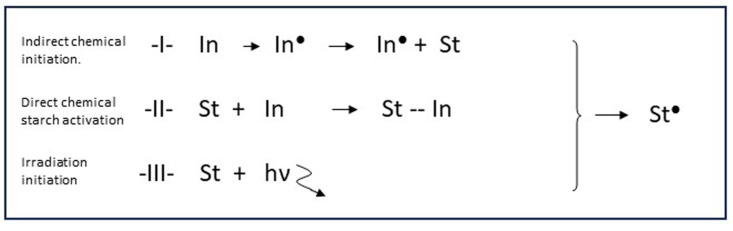
The three principal methods (class I–III) to create starch macroradicals. In = Initiation agent, St = Starch, hv 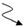 represents high energy radiation, ● = radical state.

In the reviews already mentioned [1,2,3,4], a good overview can be found of the huge variety of initiation methods that have been reported throughout decades of starch grafting research. Most of the systems belong to one of three different categories, according to the principal mechanism of radical creation at the starch backbone. These three principles are listed in Figure 5 and are designated ‘class I/II/III’. The respective classes are discussed below, since they are directly related to the potential to obtain good graft selectivity. Also, aspects related to use in larger-scale reactors are briefly assessed. 

#### 4.1.1. Class I: Activation by Radicals Created at Random in the Solution 

This is the class of initiation systems which produce radicals throughout the reaction solution. Redox reactions like Fenton’s as used in our work [8] are an example of this class, as are agents that form radicals by thermal dissociation. Examples of the latter are potassium and ammonium persulfates (KPS, APS) and organic peroxides like butyl or benzoyl peroxide [1]. All of these systems create radicals more or less at random in the typically used aqueous solution, after which the radical activity is transferred to the starch backbone. As already mentioned in Section 1 and Section 3.1, this can be designated as indirect initiation. Because of the random nature and delocalized creation of radicals, homopolymer formation is inevitably associated with indirect initiation. This class of initiation system is probably the easiest to scale up. 

#### 4.1.2. Class II: Radicals via a Direct Reaction with Starch

A second class defines initiation agents that also show a redox reaction but, in this case, starch-OH groups are the reducing part of the redox couple. Cerium(IV) ammonium nitrate (CAN) is used in many graft research projects [1,2,4]. Other examples include manganese or other transition metal ion systems [14]. The reaction mechanism of cerium(IV) initiation via the formation of an intermediate complex with starch is clearly described in many literature sources, e.g., [1,3,4]. In principle, such systems are more selective towards grafting reactions since the polymerization starts from a starch-bound radical. In Liu et al. [15] and in other older literature, it is stated that cerium is expensive, but that information appears to be outdated. Still, a major disadvantage of the CAN system is that it needs strong acidic conditions that can degrade the starch backbone [10]. Also, the complex formation with starch is a slow process [4,16]. There is some evidence that the promise of high selectivity is not always obtained [16,17]. The removal of residual cerium from the product after the reaction is never mentioned in the literature, but perhaps it can be tolerated in most applications. One aspect of graft initiations that is also hardly ever addressed in the literature is a low initiator efficiency. This means that only a small fraction of the radicals created lead to a successful propagating graft chain. From results of Okieimen et al. [18], we could estimate that, for Ce(IV)-initiated grafting of ethyl acrylate, an initiator efficiency in the order of 0.001, or 0.1%, is achieved. Calculation examples are in the Supplementary Information. A better but still low initiator efficiency of <1% was calculated from a paper of In-Hwan Park et al. [19], for ammonium persulfate/sodium metabisulfite initiation of acrylic acid grafting. These numbers are in sharp contrast to industrial polymerization processes, where initiator efficiency figures of 30–80% are the standard [12]. When the cost of initiator is a factor of importance in process economics, this aspect must be weighed as well. Still, for the reasons mentioned we consider CAN initiation, in spite of its selectivity potential, more suitable for laboratory-scale studies than for larger-scale industrial operation.

#### 4.1.3. Class III: Activation by High-Energy or Microwave Irradiation 

Irradiation as a method of starch activation has been investigated by several authors, as listed in an overview by Meimoun et al. [1]. Starch activation by high-energy irradiation was already studied extensively by Reyes et al. [20] in 1963, see also Section 6.3. Such systems need expensive equipment, which is an economic setback. More recently, microwave initiation methods, as such or in combination with agents that create radicals by thermal dissociation, have been reported [1,2], e.g., by Singh et al. [21]. A common issue with irradiation is the depth of penetration. Microwave rays do not go deeper than a few centimeters in water. High-energy systems probably have more penetration depth. Still, insufficient or uneven distribution of radicals throughout the reactor is likely, which is a potential disadvantage for application of irradiation initiation methods in commercial-scale large tubular or tank reactors. Like with cerium salts, we consider class-III initiation methods probably more suited for lab-scale research. 

#### 4.1.4. Combinations of Initiators 

There are some reports in the literature about grafting systems in which a combination of initiators has been applied, with favorable effect on the graft selectivity. Two examples are mentioned by Meimoun et al. [1]. In one case, the addition of maleic acid to a mixture of potassium persulfate and benzoyl peroxide appeared to increase the grafting percentage from 33% to 60% [1]. Although it is not mentioned, this can be expected to be accompanied by an increase in grafting efficiency as well. In another example, the combination potassium persulfate/acetone sodium bisulfite showed a non-quantified favorable effect on GE for poly(vinyl acetate) grafting on starch [1]. More recently, Jiang et al. [22] reported on the combination of CAN and APS, two initiators of a different class according to the categories mentioned above. Interestingly, this was carried out in an extruder reactor (see also Section 6.6). CAN was added first, at the start of the reaction, and APS somewhat later. With this method, an increase in GE from 36.47% to 44.72% was achieved in the grafting of acrylamide onto corn starch [22]. 

### 4.2. Reaction Variables Like Concentrations and Temperature

Reaction variables are a major factor of influence when considering the GP and GE of the grafting reaction. The effect of the reaction variables is dependent on the type of grafting reaction system. The reviews mentioned before [1,2,3,4] give a good overview already, so here we only discuss the most important effects in relation to previous results from our own work. 

**Figure 6:**
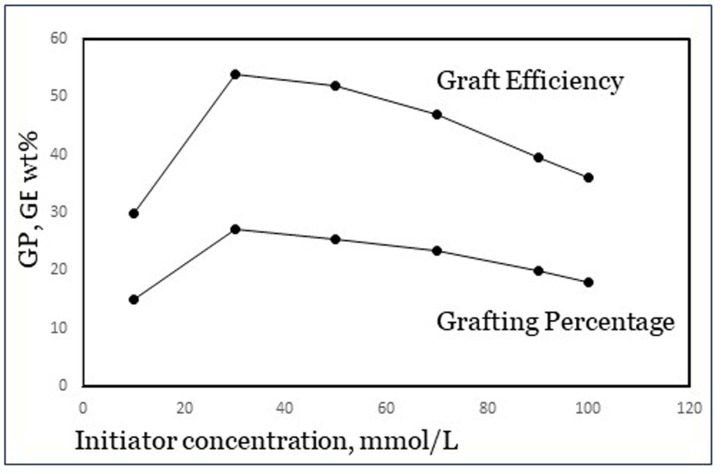
Maximum in GP and GE with initiator concentration (data from Khalil et al. [23]).

The concentration of initiator usually shows an optimum in both GP and GE. An example is shown in Figure 6, from results published by Khalil et al. [23] with potassium persulfate, a class-I initiator, and acrylamide monomer. At low initiator dosage, there are simply not enough radicals to start a polymerization reaction and so an increase in the dosage leads to improvement. However, beyond a certain amount of initiator there are too many radicals, which cause premature terminations as well as increased homopolymer formation. The reason for the latter is not fully clear, but it is speculated that in a further stage of the reaction, there may be a limited number of grafting sites available on the backbone carbohydrate, perhaps due to steric hindrance [4]. The optimum value is different for each specific grafting system including various starches, so it needs to be determined experimentally for each system. From a published experimental design study [8], we concluded that for the grafting system used in our work as mentioned in Section 2, an Fe^2+^–AGU molar ratio of 1:100 and a 1:10 molar ratio of Fe^2+^ to H_2_O_2_ are near the optimum dosage. 

In the literature, the effect of temperature shows a pattern that is similar to the influence of the initiator concentration, with the same mechanistic explanation. For our system, it was found that increasing temperature beyond 40 °C gives lower GP and GE, so 40 °C has been used in further experimental work [5,8,10].

**Figure 7:**
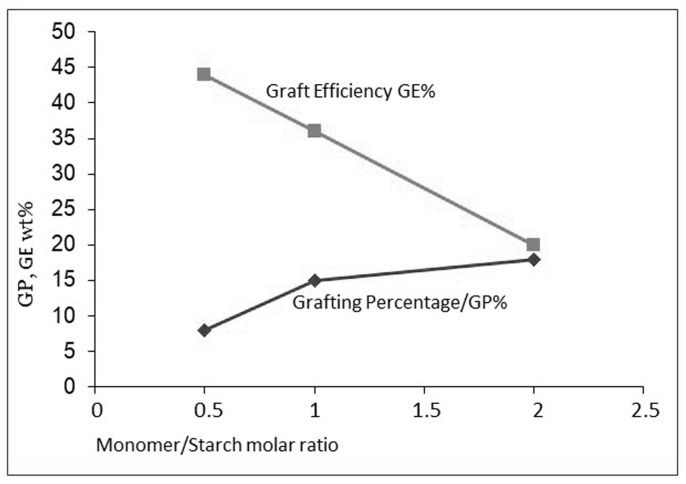
The opposite effect of M/S on GP and GE.

In the grafting system with gelatinized starch, acrylic acid, and Fenton’s initiation, the molar monomer-to-starch ratio (M/S) appears to be more important than the absolute concentrations. Unfortunately, the M/S ratio has an opposite influence on the most important result parameters GP and GE, as shown in Figure 7 (data from Witono [5]). Such a contrasting influence is seen in other starch grafting literature as well [23,24]. The optimum will be a trade-off between sufficient functional grafts for an intended application and a low extent of homopolymer production. 

### 4.3. Water Solubility of the Monomer 

Although most grafting research has been carried out with water as the solvent, the water solubility of the monomer is not often addressed as a factor of influence in published starch grafting research. However, in general reviews some valuable comparisons are made [1,4]. In Table 1, we have collected data from reviews and from various research articles. Since there are many parameters that influence graft selectivity, ranges of GE values are shown where these are available. In many graft research publications, GE values are not reported but instead reaction variables are directly linked to product properties without intermediate characterization. This table is not meant to be exhaustive but to show trends that provide a general insight into the importance of the monomer solubility in water.

Here, two effects can be clearly distinguished. With most indirect initiators (class I), there is a trend that grafting efficiency is high with hydrophobic monomers but lower with monomers that are more water-soluble. Acrylic acid, the monomer mostly used in our work, shows the lowest GE% range. The higher ends of the GE% ranges shown both for AA and for acrylamide (AAm) correspond to low monomer concentrations, which is consistent with the trend shown in Figure 7. When class-II initiation, direct starch activation, is applied there is not such a clear trend. Compared to indirect class-I initiation, higher GE% is usually seen even with water-soluble monomers. Still, values well below 100% mean that there must still be some homopolymer formed, as also discussed in Section 4.1.2. Peculiarly, some authors report no grafting at all with styrene as a monomer and CAN initiation. In the following subsection, a mechanism is suggested that provides a plausible explanation for these observations. 

## 5. A Chemical Engineering Approach to Different Grafting Systems

**Figure 8:**
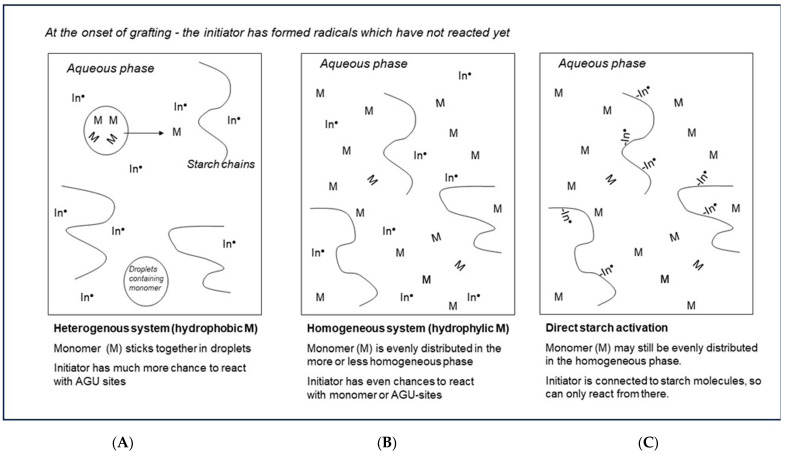
Schematic representation of different grafting reaction environments: (**A**) Monomer is poorly water soluble; (**B**) Monomer is well soluble; (**C**) Direct activation of starch only In = Initiation agent, M = monomer, ● = radical state.

Figure 8 presents ‘artist impressions’, comparing the different reaction environments according to chemical engineering principles. In Figure 8A,B, an indirect initiation agent just has started to create radicals. In the system with monomers that have low solubility in water (Figure 8A), monomer molecules will tend to stick together and form a separate discrete phase in the reactor fluid. The monomer inside these droplets cannot contact the omnipresent initiator. So, these radicals can only react with starch or with the relatively small amount of monomer at the interface or with monomer that diffused out of the discrete phase. This situation favors highly selective starch activation over homopolymer formation.

In Figure 8B, the situation is represented when the monomer is also omnipresent in the solution, as is the case with water-soluble monomers. Here, the chance that initiator radicals meet a monomer and start a homopolymer chain is much higher—in other words, the selectivity of the initiation is fundamentally lower, which is clearly reflected in the data shown in Table 1. Indeed, the monomers with the highest water solubility, AA and, to a lesser extent, also AAm, show lower ranges of GE values as compared to the other monomers. Also, the huge and contrasting influence of the M/S ratio on GP and GE as illustrated in Figure 7 can easily be understood. When there is less monomer available near the grafting site, selectivity is higher. However, when there is more monomer available, at higher M/S dosage, more grafted polymer is produced, but at the cost of lower selectivity and thus even more by-product homopolymer.

Figure 8C shows a situation where only direct activation of starch is applied (class-II initiation systems). Now, there are only a few radicals in the solution since most radicals are present at the starch backbone. Even if the monomer is omnipresent there, the radicals predominantly start a grafted polymer chain. In this situation, in theory a high graft selectivity is ensured. Indeed, in Table 1 GE data tend to be higher with CAN initiator and acrylic acid although not with AAm. Since cerium salts suffer from slow complex formation with starch, it may be that two hours of overall reaction time was too short in this case [25]. Anyway, the observation of Athawale and Rathi that with CAN initiation there is no significant effect on GE of monomer solubility or polarity [27] perfectly fits into this schematic consideration.

It is also striking to see some extremely poor grafting results with cerium salts and styrene [25,27]. Here, the very low water solubility of the monomer may be a real disadvantage. In this situation, grafting reactions can only start and propagate with the small amount of monomer that diffuses out of the discrete phase or when there is incidental contact between the active sites on the starch molecules and monomer droplets. It will be interesting to see whether the addition of a cosolvent, that will allow more of the monomer to be dissolved and come into contact with the substrate, can improve grafting in this particular case. Since our work is directed to acrylic acid, further discussion of this topic is beyond the scope of this article.

The potentially beneficial effect of initiator combinations as mentioned in Section 4.1 is quantified in the work of Jiang et al., at a 23% relative increase in GE [22]. At first CAN, a class-II initiator was added and somewhat later the class-II initiator APS. In the time between, some of the monomer will have been consumed by polymerization reactions so at the time the second initiator is inserted, there is already a lower M/S ratio. This may explain the beneficial effect on graft selectivity.

The concept of a separate phase in the reaction environment with a hydrophobic monomer is supported by observations from several other authors. Kislenko [32] reports that in the graft polymerization of methyl acrylate onto cellulose, emulsions are formed, where interface effects have large impact on the grafting results. The scheme of Figure 8A shows some superficial resemblance to emulsion polymerization, but even more to suspension polymerization [12,33]. In both cases, monomer is present in droplets as a separate phase. Without an emulsifier, hydrophobic droplets may tend to coagulate, but in suspension polymerization, just as is usual in starch grafting research, there is stirring/agitation that keeps the droplets dispersed. An analogy with emulsion polymerization is that with a monomer that has a low water solubility, the monomer concentration in the aqueous phase is always low or even near starvation. In our scheme, this means a low M/S ratio at the grafting site, which is evidently beneficial for the selectivity of the initiation. Another confirmation of the idea of monomer droplets as a separate entity in the aqueous reaction solution is found in results from De Graaf [26], in earlier work in our laboratory. It was shown that in a bench-scale extruder reactor, an increase in the screw speed leads to better grafting results of styrene onto gelatinized starch. Since an increase in the screw speed leads to more intense micromixing, this can be explained as follows. At a higher mixing rate, droplets of a separate phase will be smaller and diffusion through the larger interface will be increased. The improved mass transfer was shown to have a positive effect on the grafting results, even when the residence time and thus the reaction time are shorter at higher screw speed [26]. This observation only emphasizes the importance of phase-interface effects, with the strongly hydrophobic styrene monomer.

So, in fact, such reaction engineering insights into the differences between various grafting systems are not really unknown yet. Still such information is only scarcely reported for starch grafting reactions, and it is scattered over the vast literature on the topic of starch grafting. We think that the added value of the present considerations is that the various mechanisms both at the molecular scale and at the scale of the reactor are shown in an imaginative graphical and comprehensive manner, for the first time. The large impact of water solubility of the monomers combined with the choice of the class of initiator is clearly explained here. From these considerations, it must be concluded that the system of our focus, with the water-soluble monomer acrylic acid and indirect initiation with Fenton’s, unfortunately suffers from challenges with regard to obtaining good graft selectivity.

## 6. Methods to Improve the Graft Selectivity

### 6.1. General: Principal Approaches to the Selectivity Challenge

The considerations presented in Section 5 enable us to define some possible approaches to improve the graft selectivity in the acrylic-acid–Fenton’s system. In the following paragraphs, three principal approaches are discussed: (A) to improve the ‘final’ selectivity even if the initial selectivity is not optimal, which is limited to reactions with a polymerization crosslinker: Section 6.2. (B) Starch modifications or other steps prior to the start of the grafting reaction to enable more selective starch activation, to mimic the situation with direct initiation: Section 6.3. (C) To make the reaction fluid more similar to the situation with hydrophobic monomers: Section 6.4. The considerations in Section 6.2, Section 6.3 and Section 6.4 are for a large part based on results in our laboratory. This includes several new experiments as well as previously published experiments that are presented here in a coherent way. Also, there are literature reports on these issues which have been incorporated in the discussions per topic without the ambition to be exhaustive, for example, in the considerations about pre-modifications of starch.

### 6.2. Approach A: Use of a Polymerization Crosslinker

In principle, the addition of a polymerization crosslinker can increase the overall or final selectivity, the GE% that is obtained after the synthesis reactions. However, this potential remedy only applies when an application is considered that asks for a crosslinked network of polymer chains, like in superabsorbent materials. There is ample literature on the topic of superabsorbents based on starch, including books and other good reviews [7,34,35]. In these reviews, the chemistry of polymerization crosslinking has been described as well as other crosslinking options that are outside the scope of the present paper. N, N’-methylene bisacrylamide (MBAM) is the most common polymerization crosslinking agent and has also been used in our laboratory [36]. The crosslinker molecule contains two C=C groups that are incorporated into the polymer structure during the reaction, which results in a three-dimensional network. The C=C groups will most probably be equally reactive towards growing polyacrylic acid chains, regardless of whether the respective chain is attached to starch or if it is a non-grafted polyacrylic acid. Therefore, it quite imaginable that when one of the C=Cs has been taken up in a grafted chain, the other C=C group can connect to a growing chain of homopolymer. When occurring in sufficient numbers, the amount of polymer that is connected to starch is increased, in other words, a higher GE is obtained at the end of the process. Chain mobility and meeting probability are of importance, as is the amount of crosslinker used. In the literature on superabsorbents, the effect of the addition of soluble homopolymer to the grafted network is not mentioned. It is most usual that crosslinker dosage is optimized towards a compromise between a high absorption capacity and sufficient chain mobility for network swelling [7]. In the book edited by Pradan [34], there are two brief notes about insufficient crosslinking. In that case, there will be a soluble fraction in the synthesized superabsorbent material which affects the absorption capacity. The presence of a soluble fraction can only mean that some of the polymer chains have not become attached to the 3D network. Translated to the situation with grafted starch, this corresponds to homopolymer that has not been incorporated into the network of grafted polymer chains.

**Figure 9:**
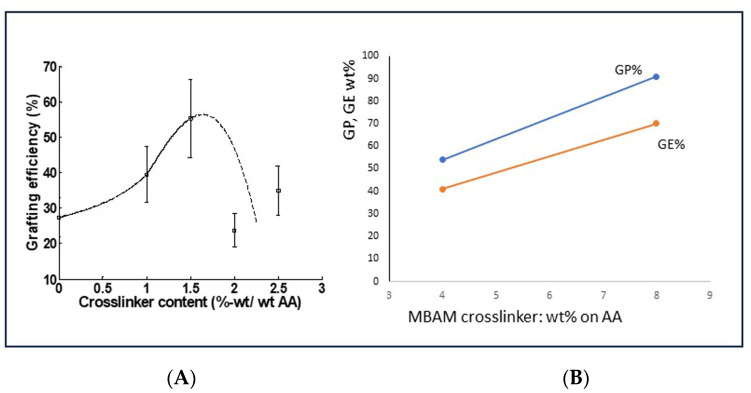
Effect of crosslinker content on GE in synthesized superabsorbent materials in our laboratory. (**A**) Data from Witono et al. [37]; (**B**) Data from more recent lab work; AA-Starch 3:1 (molar), Ammonium Persulfate (Class-I) initiator, more details are in the Supplementary Material.

A few data that were obtained in our laboratory may support the idea that the addition of a polymerization crosslinker can lead to the incorporation of non-grafted polymer chains into the grafted network. This is shown in Figure 9. The data in Figure 9A are not really convincing yet, since after an initial increase in GE%, the trend seems to be reversed. However, a few more recent data in Figure 9B show a clear positive effect of increased crosslinker dosage on the grafting result parameters GP and GE. However, this clearly needs more research. Because it is limited to applications of grafted starch in superabsorbents, this potential method to reach a higher final graft selectivity is not explored deeper here.

### 6.3. Approach B: Preceding Process Step(s) and Creation of Starch Macroradicals before Adding the Monomer

An extra process step prior to grafting makes the whole procedure of the synthesis of grafted starch more complicated and probably more expensive. An economic evaluation should be made whether a possible higher graft selectivity that may be obtained by additional process steps could be compensated by lower cost for homopolymer separation. However, such data have not been reported yet in the literature. So, in this paper we discuss the scientific aspects only. There are some interesting options to improve grafting results with preceding process steps and more selective starch activation with water-soluble monomers.

Starch gelatinization is a preceding process step which is standard for all experiments in our laboratory [5,10,36]. The potential advantages have already been discussed [5] Shortly, by gelatinization the AGU groups that are inside granules are also exposed and can take part in reactions, where internal diffusion limitations are likely to occur with starch in the granular state. After gelatinization, the starch chains are fully exposed so, in fact, the ‘effective’ concentration of AGUs is increased, which is beneficial in the competition with monomer for initiating radicals. Also, the overall rate of the reaction will be higher, and the issue of uneven distribution of grafts along the starch chains is avoided. Since we did not carry out experiments with granular starch, we cannot make a complete comparison. Data from the literature are not completely clear about this [5] but several authors confirm an advantageous effect of using gelatinized starch in a grafting process, both on grafting percentage and grafting efficiency, e.g., Park et al. [19] and Hebeish et al. [37].

Vazquez et al. [13] adsorbed Fe^2+^ ions on starch prior to adding the monomer and H_2_O_2_ in order to have radicals created in the proximity of starch. This method had limited success. A reasonable average graft selectivity of 45% was reported, but no comparison was made to the situation without pre-adsorption of the ferrous ions. This system could not overcome the controversial effect of the concentration of the monomer methacrylic acid on GE and GP, similar to what is shown in Figure 7. Why the Fe^2+^ ions should attach to starch is not mentioned in this paper. It is most likely due to the effect of negatively charged phosphate groups, known to be present in potato starch only and in a ratio of about 1 per 250 AGUs [38]. It can be imagined that inserting more phosphate groups in any starch prior to graft copolymerization may have a greater positive effect, especially with Fenton’s. There is no further literature about this idea, except one notation in a book from Hebeish and Guthrie [39]. It is mentioned there that, prior to the grafting of several monomers onto cellulose, phosphorous acid was reacted with the backbone with the intention to improve graft selectivity. Since phosphorylation is a proven modification process in the starch industry [40], it may be an interesting topic to investigate further.

Many more pre-modification methods have been tested and reported in the literature on carbohydrate grafting. A complete overview is beyond the scope of this article. However, since in most cases a first report was never followed up, perhaps these modifications were either unsuccessful or produced unwanted by-products like methods involving halogens (e.g., [37]). Another reason may be that starch integrity was affected too much, as mentioned by Wilham et al. [41], in substituting starch with allyl groups.

**Figure 10:**
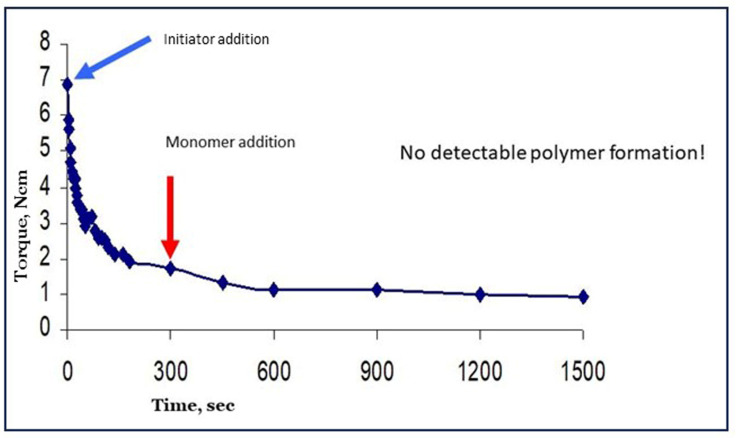
Disappointing results from pre-initiation with Fenton’s and cassava starch.

Reyes et al. have published interesting papers on the creation of radicals in dry starch by irradiation with γ-rays from ^60^Co, a radioactive isotope [20,42]. When the activated starch was put in contact with an aqueous solution with acrylic acid monomer, starch became grafted with almost 100% selectivity. This is by far the best result ever reported. Also, these results indicated that water does not function as a chain-transfer agent for starch-bound radicals since, apparently, no homopolymer was found in this system. However, despite a promising pilot scale study [42], the method never resulted in a real process as far as we know. The application of high-energy radiation may have been the bottleneck, both for economic and general safety reasons. Other methods based on chemical pre-initiation, but with only mixed success, have appeared in the literature as listed in [10]. In that paper, we also reported on an attempt to apply pre-initiation with Fenton’s reagent. The results were really disappointing since the viscosity of the gel as reflected in the registered torque values very rapidly deteriorated, as shown in Figure 10 (data from earlier work [5,10]). Oxidative degradation of the starch into smaller fragments is the most plausible reason for the observed loss of viscosity [10]. Since high viscosity or long intact chains of starch are the basis for most applications of grafted starches, we considered this method of pre-initiation not useful for further investigation.

To conclude, perhaps some pre-initiation or pre-modification methods with starch could be feasible and worth investigating further, but Fenton’s reagent was unsuccessful.

### 6.4. Approach C: Keeping the Monomer-to-Starch Ratio Low, by Dedicated Dosage of Monomer

**Figure 11:**
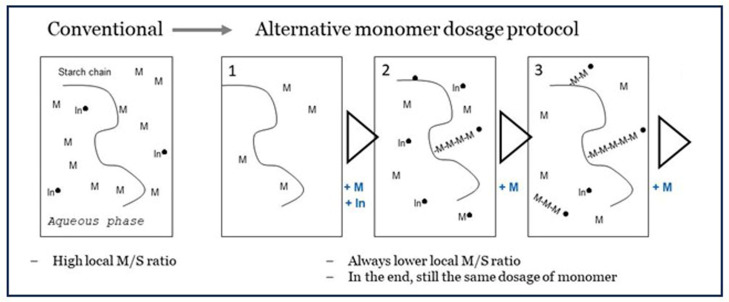
The principle of dedicated dosage, versus the more conventional method, all monomer directly at the start of the reaction. In = Initiation agent, M = monomer, -M-M-M- = growing polymer chain, ● = radical state.

The disappointing results of pre-initiation with Fenton’s show that there must always be some monomer in the system at the moment of radical generation, else the OH^•^ will affect the starch backbone. Then, a logical idea is to divide monomer addition into several smaller portions that are subsequently fed to the batch reactor (shown in Figure 1). The addition intervals cannot be too long to ensure there is no monomer depletion but not too short that the effect of low M/S ratio at the grafting sites is overruled. In exploratory experiments, we tested five equal portions. The first is mixed in before the initiator is added followed by four portions added in five-minute intervals. The principle of this ‘dedicated dosage’ is shown in Figure 11. The full set of experimental results is presented in Table 2. There, results from the method of adding monomer acrylic acid in five portions in intervals of 5 min are compared to results with the conventional method of adding all of the monomer before the initiator. For each condition, there are three measurements on different dates over a period of several months. Also, the experiments were carried out by different students. Nevertheless, the moderate standard deviation (StDev) shows that the differences between the dosage methods are significant and reliable. More details, including on the calculation methods, can be found in the Supplementary Materials.

The method of adding monomer in portions over time shows a clear improvement of both grafting percentage (17 => 28 wt%) and graft selectivity (19 => 31%). With the conventional method, a graft selectivity of over 30% could only be obtained at lower total dosage of monomer. In this situation, GP was also lower as already shown in Figure 7. This can only mean that the method of dedicated dosage has principally broken the reverse effect and the trade-off between GP and GE, which is characteristic for graft copolymerization with water-soluble monomers. While a positive development, a GE of 31% is still far from the values that are seen when grafting with hydrophobic monomers, as the data in Table 1 point out. So, there is ample room to further improve the method of dedicated dosage, for example, by varying the size of the respective portions and the time intervals. It will be most optimal if monomer is supplied at the moment when the earlier portion becomes depleted. It is also imaginable that feeding the monomer slowly in a continuous fashion, at the rate of its disappearance, is even more optimal. Such a refinement of a dedicated dosage method needs much more experimental effort, perhaps even a completely new research project, so this is still in the future. For now, we have at least demonstrated that the principle of keeping the monomer-to-starch ratio low at the start of the reaction, while in the end still having added the full amount of monomer, is valid since it leads to better grafting results with the water-soluble monomer acrylic acid.

With other polymerization systems, it has been reported in the literature that the dosage rate of monomer is a useful tool to influence the properties of the polymeric product, e.g., [43]. However, in starch graft polymerization research it is seldom reported. The paper of Trimnell and Stout [44] is a rare example of such an earlier application of a method of slow dosage. They show a good grafting efficiency, up to 59%, but with a much lower total dosage of the monomer acrylic acid. In that work, M/S is four times lower than in our standard experimental operating conditions. A direct comparison with the situation of addition of all of the monomer at the start of the reaction is not made. Also, the use of UV-irradiation initiation and oxidized starch as the backbone makes those results difficult to compare with ours. Brockway [45] has added monomer in portions but together with portions of initiator (Fenton’s). No appreciable effect on graft selectivity is seen here. However, this work is also not really comparable with our system since the addition of fresh initiator with every portion of monomer might well increase the chance of new homopolymer being formed. The main intention of that work was to increase the frequency of grafting, which was indeed achieved, as also reported in our previous review paper [6].

### 6.5. Concluding Considerations about Methods to Improve the Graft Selectivity

Method A, to improve the final selectivity by crosslinking chains, seems to have a beneficial effect on both GP and GE, but this approach is limited to grafting systems directed to synthesizing superabsorbent materials. Method B, with pre-modifications of starch, may work to some extent. Most promising in this respect is, perhaps, the idea to have initiator adsorbed on the starch before the start of the grafting reactions. To exploit this potential probably also means an additional modification of the starch. Generally, whether the addition of a more complex process step, other than pre-gelatinization, is viable in terms of process economics is uncertain and will need to be explored further. Pre-initiation may work for other grafting systems and with other initiation methods, but pre-initiation with Fenton’s proved unsuccessful. From the methods tested in our laboratory, method C, the addition of monomer in portions in time intervals to a batch reactor, appears to be the most promising option. This approach is not limited to one specific application of grafted starch. So far, it has been demonstrated that by applying a low monomer-to-starch ratio throughout the reaction, the trade-off between GP and GE that we found before is principally broken. Since a graft selectivity of 31% is not really optimal yet, the method needs to be refined further. This can be considered as a lead for future research work. That may also involve tests with combinations of initiators.

### 6.6. An Outlook towards Application of the Low M/S Principle in a Large-Scale Continuous Process

It would be an interesting chemical engineering challenge to implement the principle of keeping the monomer concentration low during a starch grafting reaction at a larger scale in a continuous reactor. The first choice is between a continuous stirred tank reactor (CSTR) and a plug flow reactor (PR). In a CSTR, the feed stream is immediately mixed with the overall contents of the reactor, where the conversion of monomer has already progressed to or is near depletion. So, in principle this concept ensures a low monomer concentration, particularly when the reactor is operated at high conversion. However, when starch is processed in the gelatinized state, there is high viscosity already at the start of the reaction. Cassava starch may be easier to process in the gelatinized state since, due to a relatively high amylopectin content, it forms softer gels than most other starches [5,46]. However, as we reported before [47], upon graft polymerization there can be an increase in the initial viscosity of a factor of 3–4 during the process. It would require an uneconomic low starch load to keep such a mixture stirrable. In industry, for the same reason, starch is more usually processed in the granular state, thus as a slurry [40]. In principle, it is possible to perform grafting reactions with granular starch, but this has other disadvantages like lower reaction rate and uneven distribution of grafts, as already mentioned in Section 6.3. It was also stated there that with gelatinized starch, better graft selectivity is expected. For these reasons, a process with starch in the gelatinized state is preferable.

There are several other flow reactors that have been proven able to handle high-viscosity fluids. In our laboratory, a tube reactor with static mixing elements has been demonstrated to be suitable for hydroxypropylation of gelatinized starch [48], a concept that has been patented [49]. This reactor shows nearly perfect plug flow. There are several more literature sources that report the use of extruders for starch grafting. The example of a bench-scale extruder in the work of de Graaf in our laboratory was mentioned before [26]. Other examples are in the work of Willet and Finkenstedt who performed graft copolymerization with acrylamide in a bench-scale extruder [50], Kugler et al. who grafted several acrylic monomers in a lab-scale extruder [51], and Jiang et al. [22] who grafted acrylamide in a combination system of a twin screw and a single extruder, also at bench scale. Jiang et al. reported that their system is able to handle very high viscosity, but no data are given. Another aspect of polymerization reactions with acrylic monomers is that they are quite exothermic, for example, in the reaction enthalpy of acrylic acid polymerization, ΔH = −77 kJ/mol [52]. Therefore, any conceptual reactor or process at a larger scale needs to have the capacity to remove a substantial amount of heat, while a high viscosity does not ease this. The extruders and the static mixer reactors mentioned are promising in this respect, but they have not yet been effectively demonstrated at larger scale for starch grafting.

**Figure 12:**
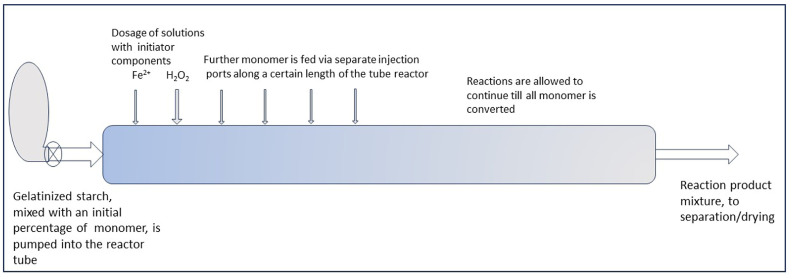
A sketch-projection of a tube reactor to perform gradual monomer dosage.

With such reactor concepts, a PR may be feasible for the graft modification of starch in the gelatinized state. The principle of keeping the M/S ratio low by monomer dosage in portions could be translated from a batch to a continuous reactor by substituting the factor time by tuning the distance between monomer injection points. Gradual dosage of monomer can then be brought about by a row of injection ports along the reactor tube, as illustrated by the sketch in Figure 12. This is only conceptual for now, since the gradual/by portion dosing still has to be refined at the lab scale. But perhaps a continuous system may also be suited for such refinement and optimization, which can start with a small-scale reactor. At present, both refinement of the gradual monomer dosage method and upscaling are challenges for future R&D.

## 7. Conclusions

It was observed that the graft copolymerization system with acrylic acid and indirect initiation with Fenton’s poses a great challenge in obtaining good graft selectivity. An opposite trend between GP and GE with increased monomer-to-starch ratio is characteristic for this system. A comparison of literature data shows that grafting with hydrophobic monomers and/or with direct starch activation usually has a better performance. The mechanism behind this difference can be found in considerations based on chemical engineering principles, described here. From these considerations, methods to also improve the graft selectivity in the AA–Fenton’s system are devised. Exploratory laboratory experiments show that a method of dedicated monomer dosage, in portions over time, has the potential to improve both GP and GE simultaneously. This can be considered a principal breakthrough, which, however, still needs further research and refinement. Also, the implementation of such a method in a continuous and larger-scale reactor is a further R&D challenge.

## Figures and Tables

**Table 1:** Graft selectivity (GE) values from literature, for five monomers with different water solubility and with direct versus indirect initiation systems.

Monomer	Solubility in Water	Initiator (Class)	GE% Range	Reference
Styrene (Sty)	0.3 g/L	KPS (I)	82–88%	Fanta et al. [25]
		KPS (I)	20–45%	De Graaf [26]
		CAN (II)	No grafting	Fanta et al. [25]
		CAN (II)	0%	Athawale et al. [27]
Methyl methacrylate (MAAt)	12 g/L	Fenton’s (I)	69–85%	Trimnell et al. [28]
		Fenton’s (I)	82–93%	Fanta et al. [25]
		MnTU (I)	83–85%	Gao et al. [29]
		CAN (II)	84–94%	Trimnell et al. [28]
		CAN(II)	65–80%	Fanta et al. [25]
		CAN (II)	56–94%	Okieimen et al. [17]
Acrylonitrile (AN)	73 g/L	Fenton’s (I)	39–91%	Fanta et al. [25]
		KPS (I)	34%	Khalil et al. [23]
		MnTU (I)	76–82%	Gao et al. [29]
		CAN (II)	89–97%	Fanta et al. [25]
		CAN (II)	62%	Athawale et al. [27]
Acrylamide (AAm)	2000 g/L	Fenton’s (I)	34–54%	Fanta et al. [25]
		KPS (I)	30–75%	Khalil et al. [23]
		HRP (I)	33–66%	Shogren et al. [30]
		MnTU (I)	69–71%	Gao et al. [29]
		CAN (II)	12–33%	Fanta et al. [25]
Acrylic acid (AA)	Fully soluble	Fenton’s (I)	19–36%	Fanta et al. [25]
		Fenton’s (I)	20–44%	Witono et al. [8]
		KPS (I)	10%	Khalil et al. [23]
		MnTU (I)	25–29%	Gao et al. [29]
		CAN (II)	10–48%	Fanta et al. [25]
		CAN (II)	29–87%	Okieimen et al. [31]

Fenton’s = Fe^2+^/H_2_O_2_; CAN = cerium ammonium nitrate; MnTU = manganese thiourea; KPS = potassium persulfate, HRP = horseradish peroxidase, an enzymatic system of initiation. Class of initiator systems: (I) = indirect initiation via radicals in the solution and chain transfer to starch, (II) = direct activation of starch. Solubility data were obtained from data sheets of manufacturing companies or general safety sheets. Further details are available in the Supplementary Materials.

**Table 2:** Results of explorations of the dedicated monomer dosage method and comparison to graft polymerization in the same conditions with conventional dosage of acrylic acid monomer.

Method	Run nr	Total M/S Molar Ratio	Results: Grafting Percentage/Efficiency	
Conventional dosage (A) = all monomer before adding the initiator	A-1 A-2 A-3 Average (StDev)	2.0 2.0 2.0	GP wt% 18% 15% 17% 17 (±1.3)%	GE wt% 20% 17% 20% 19 (±1.4)%
Monomer dosage in 20% portions, at 5 min intervals (B)	B-1 B-2 B-3 Average (StDev)	2.0 2.0 2.0	28% 26% 29% 28 (±1.3)%	32% 29% 33% 31 (±1.7)%

Other conditions: grafting of acrylic acid onto pre-gelatinized starch, T = 40 °C, starch load 7.5% in water, Fenton’s initiator, Fe^2+^:AGU molar ratio 1:100, Fe^2+^:H_2_O_2_ molar ratio 1:10. More details are provided in the Supplementary Materials and in Section 2 (standard run).

## Data Availability

The data presented in this study are available on request from the corresponding author: i.w.noordergraaf@rug.nl.

## Supplementary Materials

The following supporting information can be downloaded at: https://www.mdpi.com/article/10.3390/polym16020255/s1. The supplementary information is a Word document. It contains calculations for GE from GP as well as the data that have been used to make up Figure 4 and Figure 9B.

## References

[1] Meimoun J., Wiatz V., Saint-Loup R., Julien Parcq J., Favrelle A., Bonnet F., Zinck P. (2018). Modification of starch by graft copolymerization. Starch/Stärke.

[2] Lele V.V., Kumari S., Niju H. (2018). Syntheses, Characterization and Applications of Graft Copolymers of Sago Starch—A Review. Starch/Stärke.

[3] Athawale V.D., Rathi S.C. (1999). Graft Polymerization: Starch as a Model Substrate. J. Macromol. Sci. Rev. Macromol. Chem. Phys..

[4] Fanta G.F., Doane W.M., Wurzburg O.B. (1986). Grafted Starches. Modified Starches: Properties and Uses.

[5] Witono J.R. (2012). New Materials by Grafting of Acrylic Acid onto Cassava Starch. Ph.D. Thesis.

[6] Noordergraaf I.W., Fourie T.K., Raffa P. (2018). Free-Radical Graft Polymerization onto Starch as a Tool to Tune Properties in Relation to Potential Applications. A Review. Processes.

[7] Bucholz F.L., Graham A.T. (1998). Modern Superabsorbent Polymer Technology.

[8] Witono J.R., Noordergraaf I.W., Heeres H.J., Janssen L.P.B.M. (2012). Graft Copolymerization of Acrylic Acid to Cassava Starch—Evaluation of the Influences of Process Parameters by an Experimental Design Method. Carbohydr. Polym..

[9] Parkatzidis K., Wang H.S., Truong N.P., Anastasak A. (2020). Recent Developments and Future Challenges in Controlled Radical Polymerization: A 2020 Update. Chem.

[10] Witono J.R., Noordergraaf I.W., Heeres H.J., Janssen L.P.B.M. (2022). Torque measurement as a tool to monitor the breakdown of cassava starch gels, by the effect of Fenton’s initiator for graft copolymerization. Results Chem..

[11] Witono J.R., Marsman J.H., Noordergraaf I.W., Heeres H.J., Janssen L.P.B.M. (2013). Improved homopolymer separation to enable the application of 1H-NMR and HPLC for the determination of the reaction parameters in the graft copolymerization of acrylic acid onto starch. Carbohydr. Res..

[12] Odian G. (2004). Principle of Polymerization.

[13] Vazquez B., Goni I., Gurruchaga M., Valero M., Guzman G.M. (1989). A study of the graft copolymerization of methacrylic acid onto starch using the H_2_O_2_/Fe^++^ redox system. J. Polym. Sci..

[14] Gao J., Yu J., Wang W., Chang L., Tian R. (1998). Comparison of transition metals in the graft copolymerization of vinyl monomers to starch. J. Macromol. Sci. Pure Appl. Chem..

[15] Liu Y., Li J., Yang L., Shi Z., Deng K. (2004). Graft copolymerization of methyl methacrylate onto starch using potassium ditelluratocuprate(III). J. Macromol. Sci. Pure Appl. Chem..

[16] Gurruchaga M.B., Goni I., Vazquez B., Valero M., Guzman G.M. (1989). An Approach to the Knowledge of the Graft Polymerization of Acrylic Monomers onto Polysaccharides Using Ce (IV) as Initiator. J. Polym. Sci. Part C Polym. Lett..

[17] Okieimen F.E., Said O.B. (1989). Studies on the graft copolymerization of methyl methacrylate onto starch. Acta Polym..

[18] Okieimen F.E., Egharevba F., Jideonwo A. (1991). Graft copolymers of starch and (poly)ethyl acrylaat. Angew. Makromol. Chem..

[19] Park I.H., Song S.Y., Song B.K. (1999). Graft polymerization of acrylic acid onto corn starch in aqueous isopropanol solution. Angew. Makromol. Chem..

[20] Reyes Z., Syz M.G., Huggins M.L. (1963). Grafting acrylic acid to starch by preirradiation. J. Polym. Sci..

[21] Singh V., Tiwari A., Pandey S., Singh S.K. (2006). Microwave-accelerated synthesis and characterization of potato starchg-poly(acrylamide). Starch/Staerke.

[22] Jiang T., Chen F., Duan Q., Bao X., Jiang S., Liu H., Chen L., Yu L. (2022). Designing and application of reactive extrusion with twice initiations for graft copolymerization of acrylamide on starch. Eur. Polymer J..

[23] Khalil M.I., Mostafa K.M., Hebeish A. (1993). Graft polymerization of acrylamide onto maize starch using potassium persulfate as initiator. Angew. Makromol. Chem..

[24] Athawale V.D., Rathi S.C. (1997). Effect of chain length of the alkyl group of alkyl methacrylates on graft polymerization onto starch using ceric ammonium nitrate as initiator. Eur. Polym. J..

[25] Fanta G.F., Burr R.C., Doane W.M., Russell C.R. (1971). Influence of starch granule swelling on graft copolymer Composition. A comparison of monomers. J. Appl. Polym. Sci..

[26] De Graaf R.A. (1995). The Use of Twin Screw Extruders as Starch Modification Reactors. Ph.D. Thesis.

[27] Athawale V.D., Rathi S.C. (1997). Role and relevance of polarity and solubility of vinyl monomers in graft polymerization onto starch. React. Funct. Polym..

[28] Trimnell D., Fanta G.F., Salch J.H. (1996). Graft polymerization of methyl acrylate onto granular starch: Comparison of the Fe^2+^/H_2_O_2_ and ceric initiation systems. J. Appl. Polym. Sci..

[29] Gao J., Tian R., Zhang L. (1996). Graft copolymerization of vinylic monomers onto stach initiated by transition metal-thiourea redox systems. Chin. J. Polym. Sci..

[30] Shogren R.L., Willet J.L., Biswas A. (2009). HRP-mediated synthesis of starch-polyacrylamide grafts copolymers. Carbohydr. Polym..

[31] Okieimen F.E., Nkumah J.E., Egharevba F. (1989). Studies on the grafting of acrylic acid to starch. Eur. Polym. J..

[32] Kislenko V.N. (1999). Emulsion graft polymerization: Mechanism of formation of dispersions. Colloids Surf. A Physicochem. Eng. Asp..

[33] Khaddazh M., Gritskova I.A., Litvinenko G.I., De Souza Gomes A. (2019). An Advanced Approach on the Study of Emulsion Polymerization: Effect of the Initial Dispersion State of the System on the Reaction Mechanism, Polymerization Rate, and Size Distribution of Polymer-Monomer Particles. Polymerization.

[34] Pradan S., Mohanty S. (2023). Bio-based Superabsorbents. Recent Trends, Types, Applications and Recycling.

[35] Athawale V.D., Lele V. (2001). Recent trends in hydrogels based on starch-graft-acrylic acid: A review. Starch/Stärke.

[36] Witono J.R., Noordergraaf I.W., Heeres H.J., Janssen L.P.B.M. (2014). Water Absorption, Retention and the Swelling Characteristics of Cassava Starch Grafted with Polyacrylic Acid. Carbohydr. Polym..

[37] Hebeish A., Zahran M.K., El-Rafie M.H., El-Tahlawy K.F. (1996). Preparation and characterisation of poly(acrylic acid) starch polyblends. Polym. Polym. Compos..

[38] Van Warners A. (1992). Modification of Starch by Reaction with Ethylene Oxide. Ph.D. Thesis.

[39] Hebeish A., Guthrie J.T. (1981). The Chemistry and Technology of Cellulosic Copolymers.

[40] Bemiller J., Whistler R.L. (2009). Starch: Chemistry and Technology.

[41] Wilham C.A., McGuire T.A., Rudolphi A.S., Mehltretter C.L. (1963). Polymerization studies with allyl starch. J. Appl. Polym. Sci..

[42] Reyes Z., Clark C.F., Comas M., Russell C.R., Rist C.E. (1969). Continuous Production of Graft Copolymers of Starch with Acrylamide and Acrylic Acid by Electron Preirradiation. Nucl. Appl..

[43] Saade H., Torres S., Barrera C., Sánchez J., Garza Y. (2019). Effect of pH and Monomer Dosing Rate in the Anionic Polymerization of Ethyl Cyanoacrylate in Semicontinuous Operation. Prime Archives in Polymer Technology.

[44] Trimnell D., Stout E.I. (1980). Grafting acrylic acid onto starch and poly(vinyl alcohol) by photolysis. J. Appl. Polym. Sci..

[45] Brockway C.E. (1964). Efficiency and frequency of grafting of methylmethacrylate to granular corn starch. J. Polym. Sci..

[46] Swinkels J.J.M. (1985). Composition and Properties of Commercial Native Starches. Starch/Staerke.

[47] Witono J.R., Noordergraaf I.W., Heeres H.J., Janssen L.P.B.M. (2017). Rheological behavior of reaction mixtures during the graft copolymerization of cassava starch with acrylic acid. Polym. Eng. Sci..

[48] Lammers G., Beenackers A.A.C.M. (1994). Heat transfer and the continous production of hydroxypropyl starch in a static mixer reactor. Chem. Eng. Sci..

[49] (1994). Method of Modifying Starch.

[50] Willet J.L., Finkenstadt V.L. (2009). Comparison of Cationic and Unmodified Starches in Reactive Extrusion of Starch–Polyacrylamide Graft Copolymers. J. Polym. Environ..

[51] Kugler S., Spychaj T., Wilpiszewska K., Gor K. (2013). Starch-Graft Copolymers of N-Vinylformamide and Acrylamide Modified with Montmorillonite Manufactured by Reactive Extrusion. J. Appl. Polym. Sci..

[52] Evans A.G., Tyrrall E. (1947). Heats of polymerization of acrylic acid and derivatives. J. Polym. Sci..

