# Supplementary material for: Grafting Starch with Acrylic Acid and Fenton’s Initiator: The Selectivity Challenge
Source: Polymers (Basel). 2024 Jan 16;16(2):255. doi: 10.3390/polym16020255 (PMC10818371; doi:10.3390/polym16020255)
Supplement: Supplementary file 1 [file polymers-16-00255-s001.zip › polymers-2667267-supplementary.pdf]

Supplementary information along with:

Grafting starch with acrylic acid and Fenton's initiator: the selectivity challenge.

Inge-Willem Noordergraaf*[1], Judy R. Witono[2] and Hero J. Heeres[1]

1) Faculty of Science and Engineering, Green Chemical Engineering, Groningen University, Nijenborgh 4, 9747 AG Groningen, the Netherlands; h.j.heeres@rug.nl
2) Chemical Engineering Dept., Parahyangan Catholic Univ. Bandung, Indonesia Jl. Ciumbuleuit no. 94, Bandung 40164 – Indonesia; judy@unpar.ac.id,

* Correspondence: i.w.noordergraaf@rug.nl

Many of the data used in this paper have been reported in the PhD-Thesis of Judy Witono, Ref nr 5 from the list below the paper. Copies of the Thesis are available via the library of Groningen University and from the authors, upon reasonable request. In the Thesis and in a journal article, experimental and analytical procedures that were used in the Thesis en for the results reported in the current paper are desbribed in great detail.

GP data were derived from 1H-NMR analysis as described in Witono et al., ref 11 from the article. There and in Thesis mentioned, there are also examples of NMR-spectra, of the PAA and Cassava starch separately and of the copolymer, and also an HPLC-chromatogram.

Calculation of grafting efficiency (GE) from GP-data in Table-2, all data have been calculated according to this example.

At GP = 18%, there is 18g polyacrylic acid (PAA) on every 100 g of starch.

At a molar ratio of M/S = 2.0, there are 2 moles = 2 x 72 = 144 g of acrylic acid monomer added, to 1 mole of starch (AGU(= 162 g. So, per 100 g of starch, 88.9 g of monomer is added. It is assumed as supported by previous research (ref. Witono et al, nr 8) that all of that monomer has been converted into PAA, 100% conversion.

When 18 g of the monomer ended up in SgPAA, 18/88.9 x 100% gives the GE value, 20% (no decimals in the final value).

Experiments A1, B1 and B2 were performed by Stefan Bronswijk, A2 and B3 by Rens Koning (see Acknowlegdment). A3 is a result from Judy Witono's Thesis [5] and there GE was calculated from HPLC results.

Since these experiments were done three times, at different dates and by different persons, many months or even years apart, the very reasonable value of the Standard Deviation make that we can be confident about the resulting data. The difference between results from two conditions is much larger than StDev.

Calculation for GE in Fig.3. The original data from Judy's Thesis [7] and in [12]. This is a copy of those data, to also explain the method of calculating the GE-values across time.

For M/S = 2.0			
Time (min)	gPAA (gram)	PAA (gram)	GE = gPAA/conv monomer x 100%
5	0,05	0,61	7,4%
10	0,12	0,63	15,5
20	0,13	0,65	16
30	0,133	0,66	16,5
60	0,15	0,68	17,6
120	0,17	0,7	20%

Here, unlike after a complete reaction, the level of converted monomer was increasing during the reaction, so the value for converted monomer was obtained from adding the values of gPAA (grafted) and PAA (homopolymer).

Similarly, data for the condition M/S 1.0, GE values were calculated from data published earlier (WCCE-9 [12]).

time, min.	GE, calculated
5	30
10	40
20	42
30	42
60	40
120	39

The data for the GP and GP values in Fig.9B were calculated from gravimetry, from more recent work of Zeynab Masoumi (see Acknowlegdment). APS was used as an initiator and the molar M/S ratio was 3:1. Resulting hydrogels were first washed thoroughly with acetone to remove homopolymer and then dried in a vacuum oven.

Wg = Wh – Ws

GP = Wg/Ws x 100%

GE = Wg/Wa (under the assumption of full monomer conversion) x 100%

Starch (Ws, g)	Monomer (Wa, g)	Hydrogel (Wh, g)	Grafted PAA (Wg, g)	GP%	GE
3,83	5,00	7,32	3,49	91	70
3,82	5,00	5,89	2,07	54	41

The full study has not been published yet in an international scientific journal yet. More details of this work will appear in a future publication, so this results should be considered as preliminary.

From two papers, refered in the manuscript as 18 and 19, we could estimate initiator-efficiency values. This is only feasible if authors give values for the graft frequency, as these publications reveal.

In Okieimen (ref18), there is 40 mmol/L Ceric ions, each producing one radical. There is 2 wt% starch, or 20g/L. W/162 = 123 mMol of starch => Ce:AGU = 0.33 molar ratio. For the graft frequency, a value of 2.5/10,000 AGU is reported. So, 2.5 moles of grafted chains have been formed with 10,000 x 0.33 moles of Ceric ions = 3300 moles of initiation radicals gave 2.5 moles of grafts. This brings initiator efficiency at about 2.5/3300 = 0.08%. In a similar calculation from another entry in the table in that paper, a concentration of 7 mmol of ceric ions is stated => Ce:AGU = 0.057, this resulted in 0.8 moles of grafts per 10,000 AUG => initiator efficiency here is 0.14%. So on the average, the order of magnitude of 0.1% is mentioned in the manuscript.

Other calculations (data used from ref19) are similar to this example.
